# Gut microbiota dysbiosis and associated immune response in systemic lupus erythematosus: impact of disease and treatment

**DOI:** 10.1186/s13099-025-00683-7

**Published:** 2025-02-18

**Authors:** Aya Y. Ali, Sara A. Zahran, Mervat Eissa, Mona T. Kashef, Amal Emad Ali

**Affiliations:** 1 Microbiology & Immunology Department, Faculty of Pharmacy, Future University in Egypt, Cairo, 12311 Egypt; 2 Rheumatology & Rehabilitation Department, Faculty of Medicine, Cairo University, Cairo, 11562 Egypt; 3 Department of Microbiology and Immunology, Faculty of Pharmacy, Cairo University, Cairo, 11562 Egypt

**Keywords:** Dysbiosis, Gut microbiome, Leaky gut, Molecular mimicry, Systemic Lupus Erythematosus.

## Abstract

**Background:**

Gut microbial dysbiosis and leaky gut play a role in systemic lupus erythematosus (SLE). Geographical location and dietary habits affect the microbiome composition in diverse populations. This study explored the gut microbiome dysbiosis, leaky gut, and systemic immune response to gut bacterial consortium in patients with SLE exhibiting mild/moderate and severe disease activity.

**Methods:**

Fecal and blood samples were collected from patients with SLE and healthy volunteers. Genomic DNA was extracted from the stool samples and subjected to 16S rRNA amplicon sequencing and microbiome profiling. Additionally, enzyme-linked immunosorbent assays were employed to determine the serum lipopolysaccharide level, as an assessment of gut permeability, and the systemic immune response against gut bacteria.

**Results:**

Patients with SLE showed significantly lower gut bacterial richness and diversity, indicated by observed OTUs (56.6 vs. 74.44; *p* = 0.0289), Shannon (3.05 vs. 3.45; *p* = 0.017) and Simpson indices (0.91 vs. 0.94; *p* = 0.033). A lower Firmicutes-to-Bacteroidetes ratio (1.07 vs. 1.69; *p* = 0.01) was observed, with reduced genera such as *Ruminococcus 2* (0.003 vs. 0.026; *p* = 0.0009) and *Agathobacter* (0.003 vs. 0.012; *p* < 0.0001) and elevated *Escherichia-Shigella* (0.04 vs. 0.006; *p* < 0.0001) and *Bacteroides* (0.206 vs. 0.094; *p* = 0.033). Disease severity was associated with a higher relative abundance of *Prevotella* (0.001 vs. 0.0001; *p* = 0.04). Medication effects included lower *Romboutsia* (0.0009 vs. 0.011; *p* = 0.005) with azathioprine and higher *Prevotella* (0.003 vs. 0.0002; *p* = 0.038) with cyclophosphamide. Furthermore, categorization by prednisolone dosage revealed significantly higher relative abundances of *Slackia* (0.0007 vs. 0.00002; *p* = 0.0088), *Romboutsia* (0.009 vs. 0.002; *p* = 0.0366), and *Comamonas* (0.002 vs. 0.00007; *p* = 0.0249) in patients receiving high-dose prednisolone (> 10 mg/day). No differences in serum lipopolysaccharide levels were found, but SLE patients exhibited elevated serum gut bacterial antibody levels, suggesting a systemic immune response.

**Conclusion:**

This study confirms the gut microbiome dysbiosis in patients with SLE, influenced by disease severity and specific medication usage.

**Supplementary Information:**

The online version contains supplementary material available at 10.1186/s13099-025-00683-7.

## Background

Systemic Lupus Erythematosus (SLE) is a complex autoimmune disorder characterized by severe inflammation that damages multiple organs, primarily affecting women of childbearing age. The disease involves a dysregulated immune response and a loss of self-tolerance, which result in autoantibody production [1]. The etiology and pathogenesis of SLE remain elusive, with hormonal, environmental factors (such as drug exposure and ultraviolet light), and genetic influences [2].

The human body hosts over 10^14^ microorganisms, primarily in the gut [3, 4], with nearly 70% of total immune cells residing in the gut epithelium, lamina propria, and specialized gut-associated lymphoid tissues. Consequently, the gut microbiota and their metabolites play a crucial role in maintaining immune system homeostasis by shaping both innate and adaptive immune elements, including macrophages, toll-like receptors, innate lymphoid cells, and B- and T-cells [5].

Gut dysbiosis, or microbiota imbalance, can disrupt immune homeostasis by either activating proinflammatory cytokines or reducing anti-inflammatory cytokines. Gut microbial dysbiosis is also linked to increased gut permeability, where microbial products break the junction between epithelial cells, allowing the escape of gut commensals or gut-microbial components into the systemic circulation and consequently disturbing the immune balance. Another mechanism associated with gut dysbiosis is molecular mimicry, where commensal bacterial antigens share structural similarities or exhibit amino acid sequence homology with the host self-epitopes, potentially triggering autoimmune responses [6]. Gut dysbiosis has been linked to various autoimmune and inflammatory conditions [7–10].

In SLE, studies consistently reported reduced microbiome diversity and altered Firmicutes-to-Bacteroidetes ratio, with most studies recording significantly lower ratios [11–14]. Firmicutes produce butyrate (as a microbial metabolite), essential for Treg cells maintenance in different gut tissues [15]; its reduction has been linked to inflammatory reactions in SLE [16]. Another factor contributing to SLE pathogenesis is impaired gut permeability, commonly referred to as “Leaky Gut” [17]. The role of a leaky gut and the associated systemic translocation of gut bacteria, such as *Lactobacillus reuteri* and *Enterococcus gallinarum*, has been previously reported in SLE [5, 18].

SLE pathogenesis was also linked to molecular mimicry, where anti-double-stranded DNA (anti-dsDNA) antibodies from sera of patients with SLE have been found to react with purified *Burkholderia fungorum* cell lysate [19]. Greiling et al. (2018) identified commensal bacteria in patients with SLE with orthologs to human Ro60 autoantigen, and T-cell clones responsive to these bacteria exhibited cross-reactivity with Ro60 [20]. Moreover, C57BL/6 mice colonized with *Bacteroides thetaiotaomicron*, carrying the Ro60 version, displayed the presence of human anti-Ro60 antibodies in their blood [20]. The expansion of *Ruminococcus gnavus* in the intestine has been linked to lupus disease activity and lupus nephritis, with specific strains triggering anti-dsDNA antibody responses [21]. Notably, serum autoantibodies to native DNA serve as both a specific diagnostic criterion for SLE and a prognostic factor for lupus nephritis development [21].

The primary therapeutic approaches for SLE involve medications such as glucocorticoids, hydroxychloroquine (HCQ), azathioprine (AZA), and cyclophosphamide (CYC). Although treatment responses vary, and side effects can be significant [22–24], few studies have tried to address the impact of these medications on the gut microbial community.

The composition of the gut microbiome is known to be influenced by geographical location [25], leading to significant variations in clinical and immunological abnormalities. These variations may be attributed to genetic, hormonal, environmental, and dietary factors [2]. Limited research has focused on Egyptian patients with SLE [26, 27]. This study aimed to analyze the gut microbiome variations in female Egyptian patients with SLE using 16S rRNA sequencing, compared to an age- and sex-matched group of healthy controls. Additionally, we explored the impact of medications and disease severity on microbiome diversity. We also investigated the possible involvement of leaky gut and the systemic immune response against gut bacterial consortia in lupus pathogenesis.

## Methods

### Subjects and sampling

Fecal and blood samples were collected from patients with SLE and healthy volunteers as controls, all patients and healthy participants were adult females aged between 18 and 40. Patients with SLE were recruited from the rheumatology and rehabilitation outpatient clinic at Kasr Al-Ainy Hospital, Cairo, Egypt, between October 2020 to February 2021. Recruited patients fulfilled the 2019 European League Against Rheumatism/American College of Rheumatology classification criteria for SLE [28]. All patients had active disease status, and none were in the remission phase. Exclusion criteria included the presence of other autoimmune diseases (e.g., colitis, inflammatory bowel disease, rheumatoid arthritis, multiple sclerosis, scleroderma), intestinal conditions (e.g., irritable bowel syndrome), metabolic disorders (e.g., diabetes), infectious diseases (e.g., hepatitis, bacterial or parasitic infections), malignancies, hypertension, ischemic heart disease and pregnancy. None of the participants had taken antibiotics or probiotics or underwent surgical procedures at least a month before sampling.

Detailed histories, clinical examinations, routine laboratory tests, and immunological analyses were conducted. SLE disease activity was assessed based on Systemic Lupus Erythematosus Disease Activity Index (SELENA-SLEDAI; a modified version of the SLEDAI index). The assessment included 24 laboratory and clinical descriptors (Supplementary Table 1), giving a total score ranging from 1 to > 12. The patients were categorized according to SELENA-SLEDAI scores into the following groups: no flare (≤ 3), mild/moderate (4–12), and severe (> 12) [29, 30].

Fecal samples were collected, placed on ice, and transferred to the laboratory. Each specimen was divided into two parts: one was processed within an hour for bacterial isolation, and the other was stored at -80 ^ο^C for DNA extraction and 16S rRNA sequencing. Blood samples were collected in EDTA tubes, and centrifuged, and the serum was stored at -20 ^ο^C for further analysis.

### DNA extraction and quantification

Genomic DNA was extracted from the stool samples using QIAamp^®^ DNA Stool Mini Kit (Qiagen, Germany). The DNA was quantified using a NanoDrop spectrophotometer and a Qubit fluorometer (Thermo Fisher Scientific, Waltham, MA, USA).

### 16S rRNA amplicon sequencing

Extracted DNA was subjected to library preparation and sequencing using Illumina Miseq™ (Illumina, USA) at the National Genomics Infrastructure, Stockholm, Sweden. Paired-ends (2 × 300 bp) protocol used the universal primers (341 F and 805R) covering V3–V4 16S rRNA gene regions [31]. The library preparation process involved two PCR steps and MagSi-NGSPREP Plus beads (MDKT00010500, amsbio, USA)-based purification.

### Microbiome analysis

Microbiome analysis was performed using the nfcore/ampliseq nextflow pipeline [32]. Sequencing quality control was performed using FastQC [33], followed by read trimming with Cutadapt [34]. Amplicon Sequence Variants (ASVs) were inferred using DADA2 [35] and chimeric sequences were removed using q2-vsearch [36]. OTU-picking (operational taxonomic units based on 97% sequence similarity cutoff), taxonomic identification, and phylogenetic alignment were performed using QIIME 2 [37, 38] against the SILVA 132 bacterial database [37]. Diversity indices and plots were generated using MicrobiomeAnalyst [39].

A comprehensive analysis of the fecal microbiome was performed to evaluate differences in microbial diversity and composition between patients with SLE and healthy controls, as well as among patients with varying SLE disease severity. Further analyses examined microbiome variations in relation to medication regimens, including AZA, CYC, and HCQ, as well as prednisolone (PRED) doses stratified into two groups: low dose (≤ 10 mg/day) and high dose (> 10 mg/day). Biomarker discovery was performed using Linear Discriminant Analysis Effect Size (LEfSe). All taxonomic features were compared to detect significant shifts in microbial composition associated with disease severity, treatment, and PRED dosage.

### Gut permeability assessment

Serum LPS levels in patients and healthy controls were measured using sandwich enzyme-linked immunosorbent assay (ELISA) technique employing Human Lipopolysaccharides ELISA Kit (MyBioSource, USA). The optical density (OD) was measured at 450 nm, and LPS concentrations were determined according to a standard curve using Curve Expert 1.3 software.

### Evaluation of systemic immune responses to gut bacteria

#### Preparation of gut bacterial consortium lysates

Representative fecal samples (three from patients with severe SLE, three from patients with mild/moderate SLE, and six from healthy controls) were homogenized and cultured in brain heart infusion-supplemented (BHIS) broth (pH 7) at 37 °C under anaerobic conditions [40]. Anaerobic incubation was achieved using an Oxoid^®^ Anaerobic Jar (3.5 L HP0011A) with AnaeroGen™ environment-generating kits (Oxoid^®^, UK). Cells were harvested by centrifugation at 6000 xg for 10 min. The pellets were washed twice with 50 mM Tris-HCl (pH 7.5) and suspended in 30 mL lysis buffer containing 0.2 M NaCl, 5 mM MgCl₂, 1 mM phenylmethanesulfonyl fluoride, 1 mM Dithiothreitol, and lysozyme (1 mg/mL) [41]. Cells were disrupted by sonication and cellular debris was removed by centrifugation at 15,000 xg at 4 °C. The resulting cell lysates were collected, and protein concentrations were determined using Bradford assay [42]. These lysates served as coating antigens for detecting systemic antibodies against gut bacteria in serum samples.

#### Detection of bacterial IgG in serum

An indirect homemade ELISA was used to determine IgG antibody levels against the cell lysates from the respective gut bacterial consortium using KPL Protein Detector™ ELISA Kit (SeraCare, USA). Microwell plates were coated with 100 µL of bacterial lysates in 10x KPL coating solution at different concentrations. Serum samples served as primary antibodies targeting bacterial antigens. A two-dimensional serial dilution strategy was applied, with bacterial lysate concentrations of 1.5, 0.75, 0.375, and 0.1875 µg/mL, and serum samples at 1/20, 1/40, 1/80, and 1/160 dilutions. Each well on the plate represented a different combination of antigen-antibody concentrations. Horseradish peroxidase-labelled anti-human IgG served as a secondary antibody and was allowed to react with horseradish peroxidase substrate solution for color development. The plates were read at 405 nm and OD values were compared for SLE and healthy control groups across different tested concentrations [43].

### Statistical analysis

Various statistical tests were conducted within the QIIME2 pipeline. GraphPad Prism version 9.0 was used for statistical comparisons, with significance set at *p* < 0.05. Differences in serum LPS levels, ELISA ODs, alpha diversity indices, and taxonomic feature relative abundances were assessed using Mann-Whitney test. Biomarker discovery was performed by LEfSe which applies pairwise Wilcoxon tests with false discovery rate (FDR) correction implemented as part of its analysis pipeline [44]. Beta diversity analysis was performed by the permutational multivariate analysis of variance (PERMANOVA) statistical method, based on Bray–Curtis dissimilarity, and visualized by principal coordinate (PCoA) plots.

## Results

### Characterization of cumulative lupus manifestations

Samples were collected from 36 participants: 18 patients with SLE and 18 healthy volunteers. Immunologically, except for one individual, all patients tested positive for anti-nuclear antibodies, and 12 out of 18 tested positive for anti-dsDNA antibodies. Laboratory analysis revealed elevated serum creatinine (mean = 1.05 mg/dL) in five patients and increased 24-hour urinary protein (mean = 1.53 g) in seven; however, none had chronic kidney disease. According to the SELENA-SLEDAI score at enrollment, twelve patients had mild/moderate disease and six had severe disease.

At the time of enrollment, all patients received PRED, as a glucocorticoid, in combination with one or two additional drugs: HCQ, AZA, or CYC. HCQ and AZA were administered to 15 and 12 patients, respectively, while CYC was administered to only two patients. The clinical characteristics of the patients with SLE and the treatment protocol, including PRED doses, are provided in Supplementary Table 2.

### Microbiome analysis

Among the 36 extracted DNA from different fecal samples, five (three from patients and two from controls) did not meet quality standards for sequencing, resulting in 31 samples being analysed. After quality assessment, paired read joining, and filtration, sequencing of DNA extracted from 15 patients and 16 controls resulted in a total of 1,122,174 reads (mean = 36,199.16± 48,900.79/sample; Supplementary Table 3). Rarefaction curves showed satisfactory coverage for bacterial community analysis and comparison across different samples. All samples were rarefied to the smallest number of reads (4,413) (Fig. S1). QIIME2 identified 435 non-redundant taxonomic units, including 15 phyla, 23 classes, 35 orders, 80 families, and 282 genera.

**Fig. 1:**
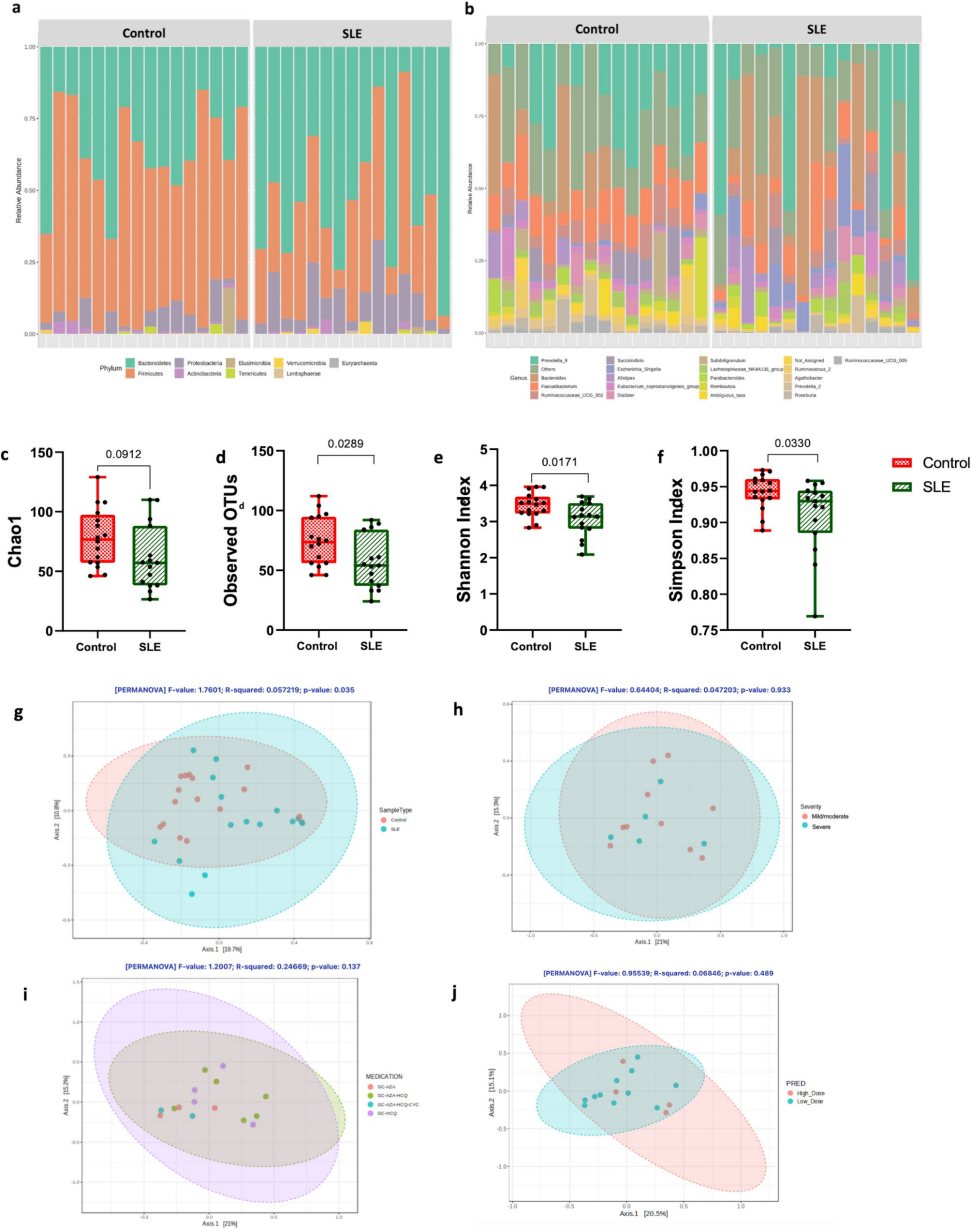
Comparison of the microbiome in patients with systemic lupus erythematosus (SLE) and healthy controls. Taxonomic summary of the microbial communities detected in the fecal samples at **(a)** the phyla level and **(b)** the top genera. The alpha diversity was estimated by different indices; **(c)** Chao1, **(d)** Observed OTUs, **(e)** Shannon, and **(f)** Simpson diversity indices. Beta diversity analysis visualized by Principal Coordinates Analysis (PCoA) of Bray-Curtis distance showing the fecal microbiome composition at the phyla level among **(g)** SLE (*n* = 15) and control (*n* = 16) groups, **(h)** mild/moderate (*n* = 10) and severe (*n* = 5) SLE subgroups, **(i)** different medication combinations, and **(j)** patients with SLE receiving low (≤ 10 mg/day) and high (˃10 mg/day) prednisolone (PRED) doses. Statistical analysis was performed using the Mann-Whitney test. *p* < 0.05 was significant. Error bars represent the standard deviation. The taxonomic summary bar plots and beta diversity plots were generated using MicrobiomeAnalyst

To enhance data quality, MicrobiomeAnalyst applied a filtration process, retaining features with a minimum prevalence of 20% and a count of at least 4. After filtering, 637 low-abundance and 21 low-variance features were removed, keeping a final set of 179 distinct features. Among these, two core phyla existed across all samples: Firmicutes (mean relative abundance = 46.37%) and Bacteroidetes (mean relative abundance = 43.28%). Other phyla, such as Actinobacteria, Lentisphaera, Proteobacteria, Tenericutes, and Verrucomicrobia, were present at lower proportions (Fig. 1a). At the genus level, 83 genera were identified, with *Prevotella_9*, *Bacteroides*, and *Faecalibacterium* as the predominant ones (Fig. 1b).

### Alpha and beta diversity analysis among groups

At the feature level, three alpha diversity indices were significantly lower in the SLE group compared to healthy controls: observed OTUs (56.6 vs. 74.44; *p* = 0.0289), Shannon index (3.05 vs. 3.45; *p* = 0.017) and Simpson index (0.91 vs. 0.94; *p* = 0.033) (Fig. 1c-f). However, alpha diversity metrics did not differ significantly based on disease severity (mild/moderate vs. severe) or among patients receiving different medications (AZA, HCQ, CYC, or varying doses of PRED; Fig. S2a-e). Differences in beta diversity between SLE and healthy controls were observed by PCoA analysis of Bray-Curtis distance, according to PERMANOVA (*p* = 0.035), indicating distinct microbial community composition (Fig. 1g). However, no significant clustering was observed among SLE severity subgroups, different treatment combinations, or varying PRED doses **(**Fig. 1h-j).

### Altered microbiota composition among different groups

Comparative analysis across all taxonomic levels between the various groups identified the potential influence of the disease status, disease severity, and treatment on gut microbial composition.

#### Disease status

**Fig. 2:**
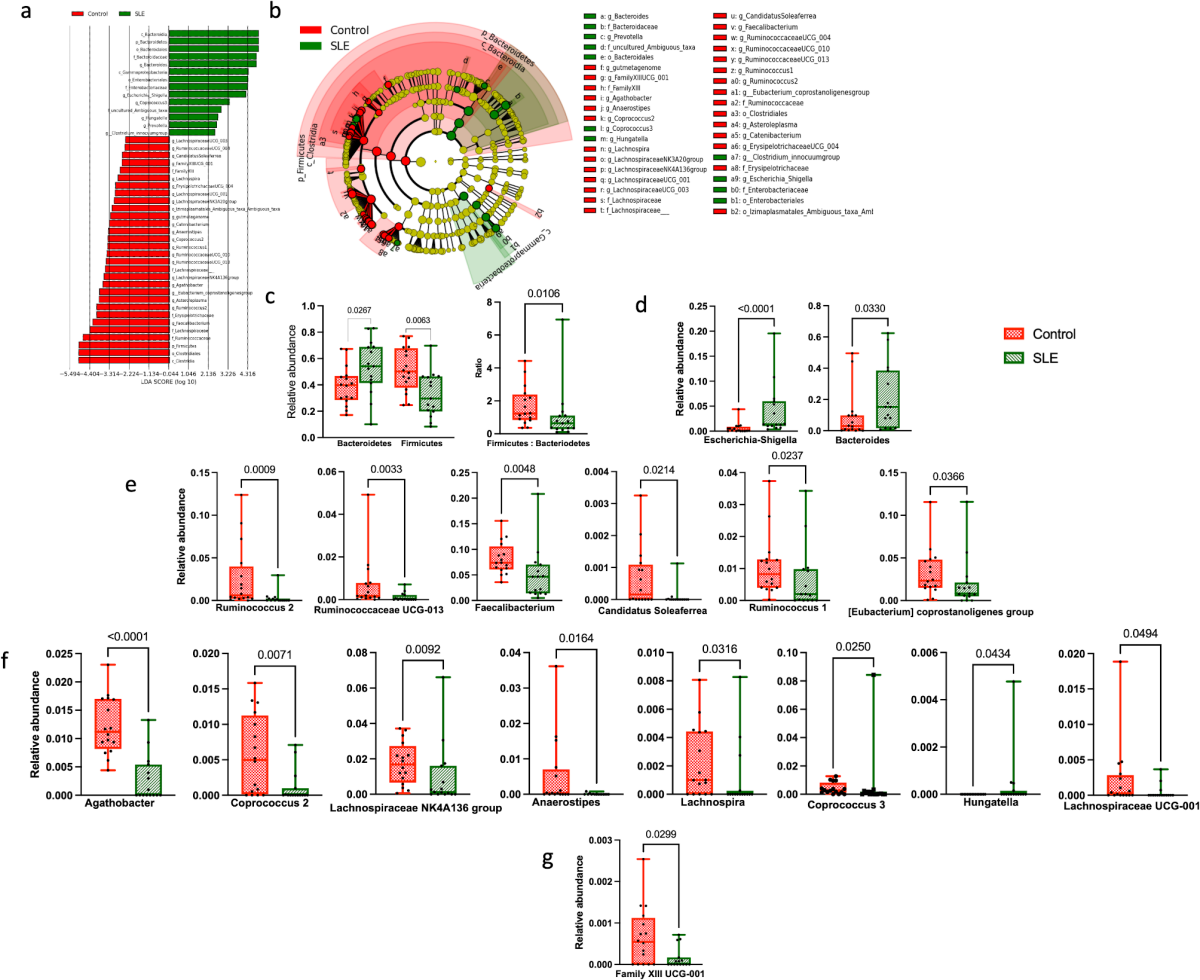
Comparative analysis of gut microbiota in patients with systemic lupus erythematosus (SLE) and healthy controls. **(a)** Histogram of the Linear discriminant analysis (LDA) scores for different abundant genera in healthy control (*n* = 16) and SLE patients (*n* = 15). Green, enriched in patients with SLE; Red, enriched in healthy controls; **(b)** Cladogram of Linear discriminant analysis effect size (LEfSe) of the microbiome of control and patients with SLE. Green and red circles are the significantly affected taxa. The diameter of each circle is proportional to the relative abundance of the taxon; **(c– g)** Boxplots representing the relative abundances of **(c)** Firmicutes, Bacteroidetes and their ratio, **(d)** Significantly elevated genera in SLE group within Enterobacteriaceae and Bacteroidaceae families, and significantly reduced genera in SLE group within **(e)** Ruminococcaceae family, **(f)** Lachnospiraceae family and **(g)** Family XIII. Statistical analysis was performed using the Mann-Whitney test. *p* < 0.05 was significant. Error bars represent the standard deviation

LEfSe identified 44 significant microbial markers between patients with SLE and healthy controls (Fig. 2a **& b**). Similar findings were recorded by the analysis performed using Mann-Whitney test at different taxonomic levels. The phylum Bacteroidetes was significantly higher (0.54 vs. 0.39; *p* = 0.0267), while phylum Firmicutes was significantly lower (0.33 vs. 0.52 *p* = 0.0063), in patients with SLE, resulting in a significantly lower Firmicutes-to-Bacteroidetes ratio (1.07 vs. 1.69; *p* = 0.01) (Fig. 2c). At the family level, SLE group showed a significantly higher relative abundance of Enterobacteriaceae (0.053 vs. 0.007; *p* = 0.0001) and Bacteroidaceae (0.21 vs. 0.094; *p* = 0.033), while Ruminococcaceae (0.16 vs. 0.28; *p* = 0.0082), Lachnospiraceae (0.06 vs. 0.11; *p* = 0.0006), and Family XIII (0.001 vs. 0.002; *p* = 0.0259) were significantly lower in abundance. Within families Enterobacteriaceae and Bacteroidaceae, only one genus in each was significantly higher in SLE group; *Escherichia-shigella* (0.04 vs. 0.006; *p* < 0.0001) and *Bacteroides* (0.206 vs. 0.094; *p* = 0.033, Fig. 2d). Several genera within Ruminococcaceae were significantly lower in SLE group, including *Ruminococcus 2* (0.003 vs. 0.026; *p* = 0.0009), *Ruminococcaceae UCG-013* (0.001 vs. 0.007; *p* = 0.0033), *Faecalibacterium* (0.049 vs. 0.082; *p* = 0.0048), *Candidatus Soleaferrea* (0.00008 vs. 0.0007; *p* = 0.0214), *Ruminococcus* 1 (0.006 vs. 0.01; *p* = 0.0237) and *[Eubacterium] coprostanoligenes group* (0.02 vs. 0.03; *p* = 0.0366, Fig. 2e). Similarly, Lachnospiraceae had several significantly lower genera, including *Agathobacter* (0.003 vs. 0.012; *p* < 0.0001), *Coprococcus 2* (0.001 vs. 0.006; *p* = 0.0071), *Lachnospiraceae NK4A136 group* (0.009 vs. 0.02; *p* = 0.0092), *Anaerostipes* (0.00008 vs. 0.005; *p* = 0.016), *Coprococcus 3* (0.0005 vs. 0.003; *p* = 0.025), *Lachnospira* (0.001 vs. 0.002; *p* = 0.0316), *Lachnospiraceae UCG-001* (0.0004 vs. 0.002; *p* = 0.049), and *Hungatella* (*p* = 0.043) (Fig. 2f). However, genus *Family XIII UCG-001* was the only member of Family XIII with significantly lower abundance in patients with SLE (0.0002 vs. 0.0007; *p* = 0.0299, Fig. 2g).

#### Disease severity

**Fig. 3:**
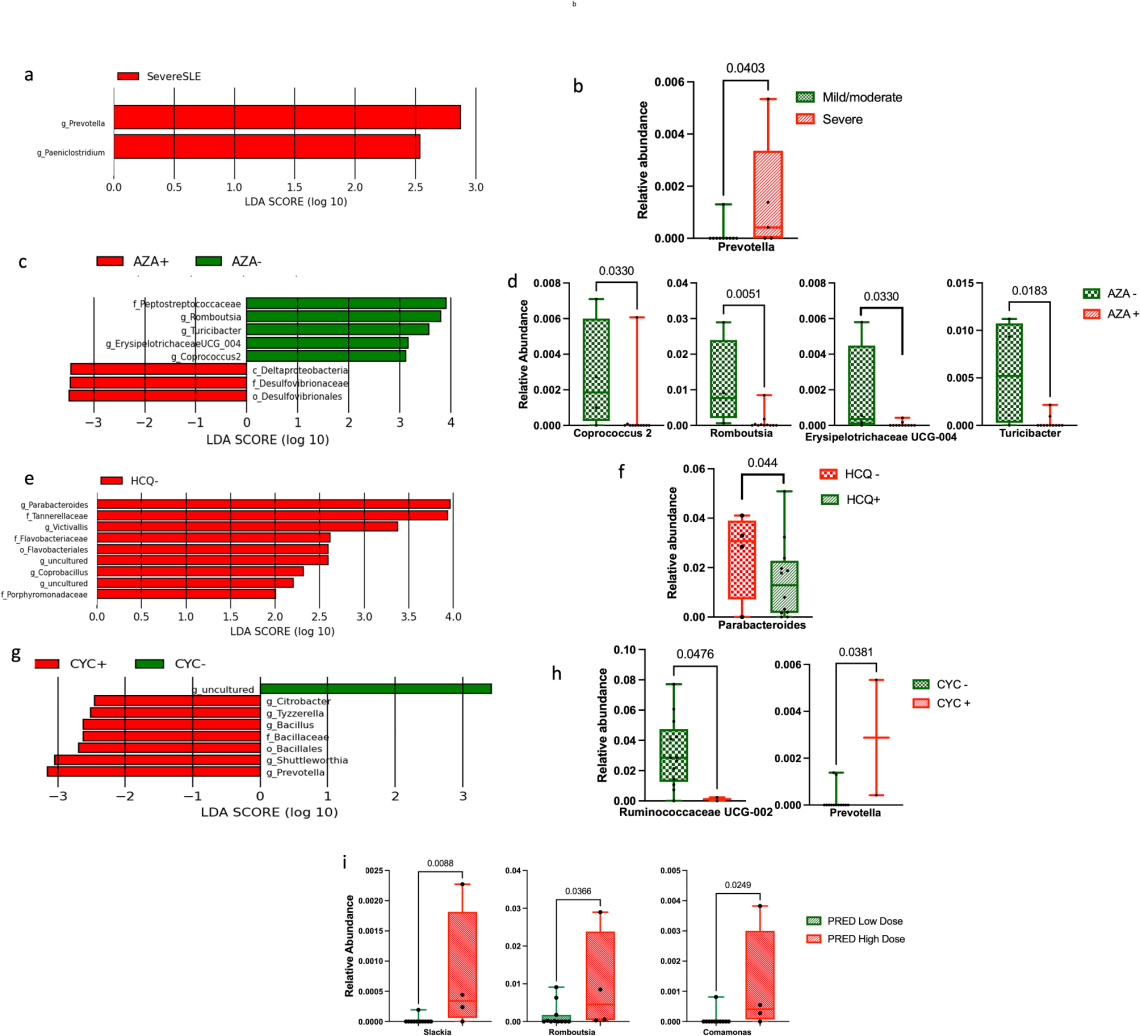
Gut microbiota in patients with systemic lupus erythematosus (SLE) with different disease severity and treatments. **(a)** Histogram of the Linear discriminant analysis (LDA) scores for different abundant genera in patients with mild/moderate (*n* = 10) and severe (*n* = 5) SLE. Red, enriched in patients with severe SLE; **(b)** Boxplot representing the relative abundances of the only significantly elevated genus in the severe SLE group; **(c)** Histogram of the LDA scores for different abundant genera in azathioprine-treated (AZA+, *n* = 11) and untreated (AZA-, *n* = 4) groups. Green, enriched in AZA- group; Red, enriched in AZA + group; **(d)** Boxplots representing the relative abundances of significantly reduced genera in AZA + group, **(e)** Histogram of the LDA scores for different abundant genera in hydroxychloroquine-treated (HCQ+, *n* = 12) and untreated (HCQ-, *n* = 3) groups. Red, enriched in HCQ- group; **(f)** Boxplots representing the relative abundances of significantly affected genus in HCQ + and HCQ- groups; **(g)** Histogram of the LDA scores for different abundant genera in cyclophosphamide-treated (CYC+, *n* = 2) and untreated (CYC-, *n* = 13) groups. Green, enriched in CYC- group; Red, enriched in CYC + group; **(h)** Boxplots representing the relative abundances of significantly affected genera among CYC + and CYC- groups; **(i)** Boxplots representing the relative abundances of significantly affected genera among groups receiving low (≤ 10 mg/day) and high (˃10 mg/day) prednisolone (PRED) doses. Statistical analysis was performed using the Mann-Whitney test. *p* < 0.05 was significant. Error bars represent the standard deviation

LEfSe identified *Prevotella* and *Paeniclostridium* as potential biomarkers for severe SLE (Fig. 3a and Fig. S3a). However, only *Prevotella* was significantly more abundant in patients with severe SLE (0.001 vs. 0.0001) using Mann-Whitney test (*p* = 0.0403, Fig. 3b).

#### Treatment groups

In AZA-treated patients, LEfSe identified eight significant taxa (Fig. 3c and Fig. S3b), with four genera from the phylum Firmicutes showing lower abundance as determined by the Mann-Whitney test: *Romboutsia* (0.0009 vs. 0.011; *p* = 0.0051), *Coprococcus* 2 (0.0006 vs. 0.003; *p* = 0.033), *Turicibacter* (0.0003 vs. 0.005; *p* = 0.0183) and *Erysipelotrichaceae UCG004* (0.00005 vs. 0.002; *p* = 0.033) (Fig. 3d**)**. In the HCQ-treated group, LEfSe analysis revealed nine significantly lower taxa **(**Fig. 3e, Fig. S3c). However, Mann-Whitney test results indicated that only *Parabacteroides* (0.01 vs. 0.03; *p* = 0.044) was significantly reduced in the HCQ-treated group **(**Fig. 3f**).** In CYC-treated patients, seven potential biomarkers were identified by LEfSe analysis **(**Fig. 3g, Fig. S3d). However, the Mann-Whitney test showed that *Prevotella* was significantly higher (0.003 vs. 0.0002; *p* = 0.0381), while *Ruminococcaceae UCG-002* was significantly lower (0.001 vs. 0.03; *p* = 0.0476) in this group **(**Fig. 3h**)**. When patients were stratified by PRED dosage into low-dose (≤ 10 mg/day) and high-dose (> 10 mg/day) groups, no significant biomarkers were detected using LEfSe analysis. However, the Mann-Whitney test revealed that the high-dose group exhibited significantly higher relative abundances of *Slackia* (0.0007 vs. 0.00002; *p* = 0.0088), *Romboutsia* (0.009 vs. 0.002; *p* = 0.0366), and *Comamonas* (0.002 vs. 0.00007; *p* = 0.0249) (Fig. 3i).

### Gut permeability is unaffected by disease status or severity

**Fig. 4:**
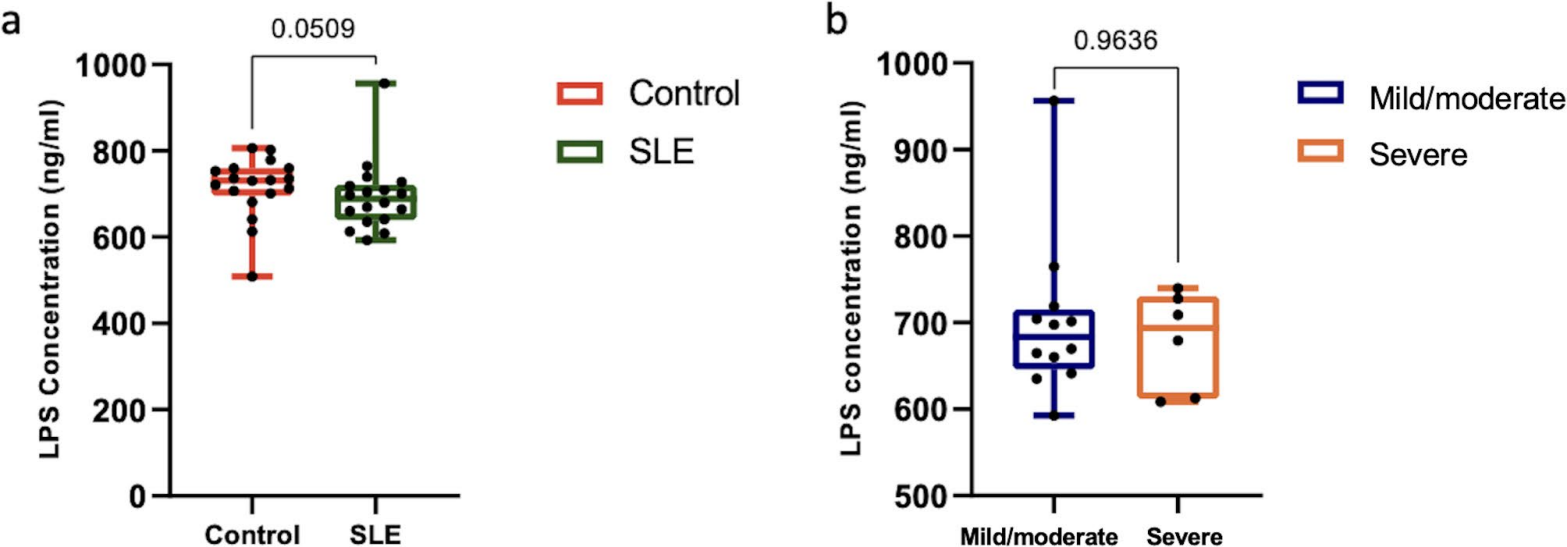
Disease status and severity did not affect the serum lipopolysaccharide (LPS) concentration. Comparative analysis of lipopolysaccharide concentration in the serum of **(a)** Patients with systemic lupus erythematosus (SLE) and healthy controls and **(b)** Mild/moderate and severe SLE groups. Statistical analysis was performed using the Mann-Whitney test. *p* < 0.05 was significant. Error bars represent the standard deviation

Serum LPS levels were not significantly different between the SLE and healthy control groups (*p* = 0.509, Fig. 4a) or between mild/moderate and severe SLE subgroups (*p* = 0.9636, Fig. 4b).

### Patients with SLE had a higher systemic immune response to gut bacteria

**Fig. 5:**
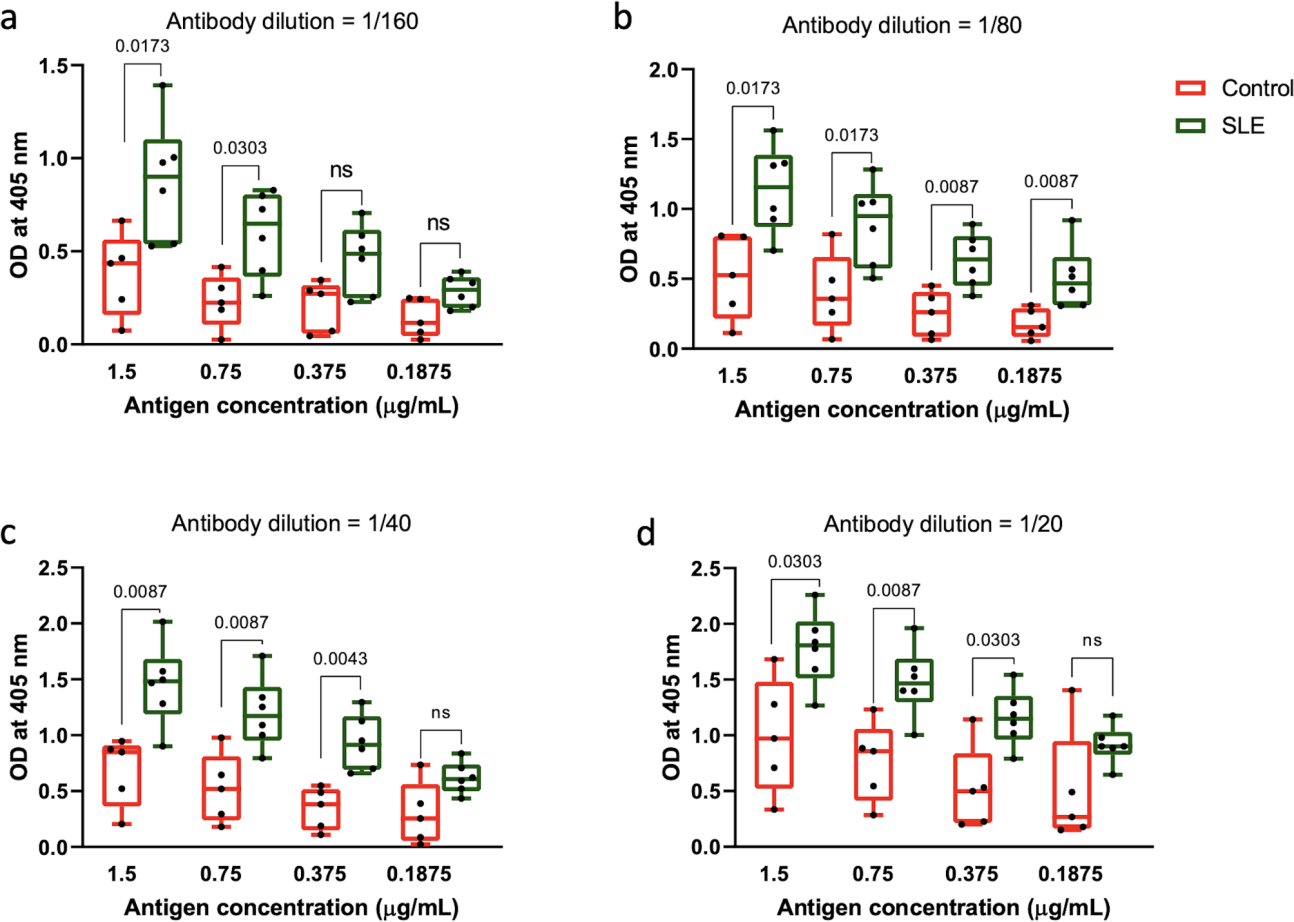
Patients with systemic lupus erythematosus had a higher systemic immune response to gut bacteria. Serum IgG response to gut bacterial lysates determined by Enzyme-Linked Immunosorbent Assay (ELISA) using combinations of different concentrations of gut bacterial lysates as antigen (1.5, 0.75, 0.375 and 0.1875 µg/mL) and different dilutions of the respective participants’ serum as antibody [**(a)** 1/160, **(b)** 1/80, **(c)** 1/40, and **(d)** 1/20]. Statistical analysis was performed using the Mann-Whitney test. *p* < 0.05 was significant. Error bars represent the standard deviation

The systemic antibody response to the gut consortium was evaluated by a homemade ELISA. The OD values were measured across different combinations of antigen (gut bacterial lysate)-antibody (respective participant’s serum) concentrations in both the SLE group (*n* = 6) and the control group (*n* = 5). Significantly higher OD values were observed in the SLE group compared to the control group across different antigen-antibody combinations. However, at the lowest tested antigen concentration (0.1875 µg/mL), no significant differences were observed between the SLE and control groups, except at an antibody dilution of 1/80. Also, no significant differences were detected at the antigen-antibody combination of 0.375 µg/mL and 1/160 dilution (Fig. 5).

## Discussion

This study explored the variations of the gut microbiome composition in Egyptian patients with SLE and its possible role in the development of systemic immunological response. The study presents a cohort of female patients aged 18 to 40, all diagnosed with SLE. Immunologically, the majority exhibited typical markers such as anti-nuclear antibodies, and anti-dsDNA antibodies, as well as other specific antibodies like ACL IgM, ACL IgG, and APA. The detection of anti-nuclear and anti-dsDNA antibodies is particularly reliable in diagnosing SLE due to their high sensitivity [45]. Laboratory analysis revealed diverse abnormalities reflecting systemic involvement, including hematological disturbances, complement deficiencies, and renal dysfunction. Clinically, patients presented a spectrum of symptoms affecting various organ systems, including the skin, mucosa, joints, kidneys, and the nervous system. SLEDAI scores varied, indicating different levels of disease severity. Treatment primarily involved glucocorticoids and HCQ, with some patients also receiving immunosuppressive agents such as AZA, and CYC. The immunological, laboratory, and medication data of the study participants align with findings from other studies on SLE [1, 13, 46].

In this study, the composition of the gut microbiome in patients with SLE was compared to that of healthy individuals. Patients with SLE exhibited significantly lower bacterial richness and diversity, as evidenced by lower OTUs, Shannon and Simpson diversity indices. Additionally, separate clustering patterns of SLE and control samples in PCoA analysis highlighted the distinct compositional differences in their microbial communities. These findings align with prior studies [1, 12, 47], providing further confirmation of the gut microbiome dysbiosis in patients with SLE. However, neither the disease severity nor the use of different medications had a notable effect on any of the alpha diversity indices within the compared groups. Similarly, previous studies reported the absence of a significant relationship between different treatments [4] or disease severity [48] and the diversity of the gut microbiomes in SLE. However, Azzouz et al. declared that lower diversity was observed in patients with SLE of high disease activity compared to those with low disease activity [21]. In contrast to our findings, Chen et al. reported higher microbiome diversity and richness in rheumatoid arthritis patients treated with HCQ [49].

To investigate the potential impact of glucocorticoids, we categorized patients receiving PRED into two groups: those taking ≤ 10 mg/day (low dose) and those taking > 10 mg/day (high dose). This classification was based on previous studies highlighting the dose-dependent effects of glucocorticoids on the gut microbiota composition. Xiang and colleagues (2022) meta-analysis reported that PRED doses up to 10 mg/day affected Chao1 index compared to healthy controls [50]. Additionally, Guo et al. (2020) recorded restoration of the gut microbiome in SLE patients receiving high doses of glucocorticoids (up to 20 mg/day) [14]. Our results, however, indicated that different PRED doses did not affect any of the alpha diversity indices.

A significantly lower Firmicutes (32.7% vs. 51.6%) and higher Bacteroidetes (53.6% vs. 39.2%) predominance were observed in SLE, resulting in a lower Firmicutes-to-Bacteroidetes ratio (1.08 vs. 1.69 in healthy controls). These findings align with previous studies from Egypt and worldwide, suggesting a characteristic pattern of lower Firmicutes-to-Bacteroidetes ratio in SLE [13, 14, 26, 46, 47]. However, few studies reported no significant alteration in Firmicutes-to-Bacteroidetes ratio in SLE, possibly due to the differences in patient demographics and lupus disease manifestations [1, 48]. Firmicutes and Bacteroidetes are the most abundant phyla in the human gut microbiota, and shifts in their ratio were consistently reported in different diseases, confirming their association with disease physiology and dietary habits [13].

There was a notable variation between patients with SLE and controls in a range of taxa abundance, including significantly lower levels of *Ruminococcus*, *Agathobacter*, *Faecalibacterium*, *Anaerostipes*, and *Coprococcus*, all are members of the order Clostridales and phylum Firmicutes. Reduction in the abundances of these genera in SLE was previously reported [4, 12, 14]. These bacteria are well known for their beneficial contribution to a healthy gut. *Ruminococcus* is vital for dietary fiber fermentation and short-chain fatty acid production, such as butyrate, which is crucial for intestinal epithelial cells [51]. *Faecalibacterium*, comprising over 5% of the gut microbiome [52], plays a significant role in butyrate synthesis [53, 54]. *Agathobacter* and *Anaerostipes* genera, both within the Lachnospiraceae family, are known for their significant butyrate production along with other fermentation products [55, 56]. A decline in *Anaerostipes* spp. has also been observed in patients with inflammatory bowel diseases, irritable bowel syndrome, metabolic disorders, *Clostridioides difficile* infection, and infantile food allergies [57–60]. *Coprococcus* genus enhances microbial balance within the host through interactions with native microbiota, promoting anti-pathogenic effects, reinforcing the intestinal barrier, and producing antimicrobial substances [61].

Similarly, significantly lower levels of genera *Lachnospiraceae NK4A136 group*, *Lachnospiraceae* UCG-001, and *Lachnospira* (members of the family Lachnospiraceae and phylum Firmicutes) were observed in SLE. The Lachnospiraceae family is known for its beneficial effects, particularly the production of microbially derived indoles, that activate the aryl-hydrocarbon receptor, triggering IL-22 release and promoting tissue repair and homeostasis [62]. In general, the significantly lower abundance of all these beneficial bacteria in patients with SLE, in comparison to the control group, highly suggests their potential contribution to the pathogenesis of SLE, possibly through a reduction in their protective role.

Interestingly, while the phylum Proteobacteria itself did not show a significant difference, a specific class within it—Gammaproteobacteria—along with the order Enterobacteriales, family Enterobacteriaceae, and genus *Escherichia-Shigella*, exhibited significantly higher levels in patients with SLE. Some studies have reported a concomitant higher abundance in the phylum Proteobacteria together with the other related taxa [12, 47]. These findings have been previously linked to intestinal inflammation, suggesting a shared characteristic of the inflammatory response observed in patients with SLE [1, 12, 63].

It was observed that genus *Prevotella* was significantly more abundant in the SLE group and in severe compared to mild/moderate patients. Intestinal *Prevotella* colonization was reported previously to induce metabolic changes in the microbiota, reducing IL-18 production, and exacerbating intestinal inflammation, and systemic autoimmunity [64]. Some *Prevotella* strains may promote chronic inflammation, contributing to human disease [65, 66]. Elevation in gut *Prevotella* abundance was reported in patients with SLE by previous studies [14, 47]. The elevated *Prevotella* abundance observed in the severe SLE group may be influenced by the disease severity [14, 47] or contributed to a severe form of the disease.

Upon exploring the effect of prescribed medications on different taxa, we noticed that patients treated with AZA exhibited a significantly lower relative abundance of *Turicibacter*, *Romboutsia*, *Coprococcus 2*, and *Erysipelotrichaceae UCG004*. A similar lower abundance of *Coprococcus 2* and *Erysipelotrichaceae UCG004* was noted when comparing the SLE group to healthy controls. Notably, 11 of the patients with SLE received AZA treatment, suggesting that these microbial alterations may not solely be attributed to the disease itself but also to the effect of AZA treatment. HCQ-treated patients exhibited a lower abundance of family Tannerellaceae (including genus *Parabacteroides*) and family Flavonibacteriaceae. Shi et al. observed that arthritic mice treated with HCQ exhibited an expansion of *Akkermansia* and *Parabacteroides*, along with a lower abundance of *Clostridium sensustricto* cluster 1 [67]. The genus *Prevotella* was significantly higher in the CYC-treated group. However, with only two patients receiving CYC, this finding is inconclusive and may relate more to disease severity; as both patients were among the severe SLE group. Interestingly, a lower abundance of *Prevotella* was previously reported in CYC-treated mice, along with reduced *Alistipes*, *Lactobacillus*,* Rikenella*, and *lachnospiraceae_NK4A136_group* [68]. Contrarily, some studies found no effect of medications like AZA, PRED, and HCQ on the fecal microbiome composition in SLE [69, 70]. The PRED dose affected the abundance of only three genera (*Slackia*,* Romboutsia*,* and Comamonas*) that were significantly enriched in patients receiving a high dose (> 10 mg/day). Another study reported that the administration of different doses of PRED to mice with SLE led to changes in different bacterial taxa and alterations in metabolic functions [71].

Serum LPS, a representative biological marker of gut permeability and microbial translocation, induces pro-inflammatory cytokines and NF-κB activation, leading to immune activation [72]. Previous studies reported that patients with SLE having an imbalanced Firmicutes-to-Bacteroidetes ratio experience higher plasma LPS levels [1, 21, 73]. Contrary to expectations, our results did not reveal significant variations in serum LPS levels between SLE and healthy controls. The reliability of LPS as a consistent indicator of gut barrier dysfunction remains uncertain due to variations in findings across studies [74]. This lack of consistency may be attributed to the interference of various factors affecting LPS detection or its relatively short half-life [75]. Moreover, the absence of a significant change in LPS could be due to differences in affinity, production, clearance, or possibly the low molar ratio of LPS [76]. Given these limitations, Lipopolysaccharide Binding Protein (LBP) was employed as an attractive marker of gut hyperpermeability than LPS [77]. Interestingly, other studies aligned with our findings, where no significant difference in LBP was reported between SLE and control subjects [78].

We evaluated the systemic antibody response to microbial proteins by using lysates of gut bacterial consortium as antigens while testing for antibodies in corresponding serum. In a similar approach, Manukyan and colleagues (2008) used the lysates from fecal microbial isolates to study the systemic immune response in patients with SLE [79]. For bacterial growth, BHIS medium was selected as it supports the growth of most microbial species inhabiting the gut. This complex, nutrient-rich medium mimics the nutritional environment in the colon and was proven to enhance the growth of different intestinal microorganisms [40, 80]. The recorded elevated serum antibodies against gut bacterial consortium in patients with SLE suggests cross-reactivity with human autoantigens, potentially triggering autoimmunity [81]; this may result from molecular mimicry between gut bacterial antigens and autoantigens, which naturally circulate in the blood during lupus. The involvement of molecular mimicry between gut microbes and human autoantigens, in lupus pathogenesis, was reported previously with *B. fungorum* and *R. gnavus* [19–21]. However, the significantly elevated level of systemic antibodies against gut consortium observed in our study was not previously reported, suggesting possible cross-reactivity with other microbial species. Alternatively, it may be caused by gut bacterial translocation and subsequent antibody production against gut microbial consortia. Given the uncertainty about the use of LPS as a reliable marker of gut permeability, further investigation is required.

In addition, performing ELISA testing on proteins extracted directly from stool would provide a wider picture of systemic immune response to gut proteins by enabling the detection of structural microbial proteins, proteins from microbial metabolism, and proteins of non-microbial origin. Future studies are therefore required to evaluate possible systemic immune responses to whole gut proteins and to identify specific protein types that might be involved in lupus pathogenesis.

Finally, this study was designed as a pilot investigation to explore the gut microbiome dysbiosis, leaky gut, and the systemic immune response to the gut microbiome in SLE. However, several limitations should be considered. A key limitation is the small sample size, particularly within subgroups based on disease severity and treatment regimens, which may have reduced the statistical power, limiting the ability to detect subtle associations. Additionally, the cross-sectional design provides only an initial snapshot of the microbiome-disease relationship, without addressing causality or directionality. External factors, such as variations in individuals’ immune responses and diet could have introduced confounding variability, affecting the interpretation of the microbiome’s role in SLE. To address these limitations, future studies should include larger, more diverse cohorts, including untreated patient groups, controlled dietary intake, and a longitudinal design to better understand the microbiome’s impact on disease progression. Additionally, the 16S rRNA microbiome analysis used did not provide insights into strain-level differences that may contribute to SLE pathogenesis. The use of LPS as a gut permeability marker has several limitations that might affect the interpretation of the overall results. Alternative gut permeability markers, such as LBP, are recommended for more reliable assessment. Moreover, the possible role of molecular mimicry in the reported systemic immune response to the gut microbiota was not evaluated in this study, and further experimental validation is needed to confirm the possible involvement of autoantibodies in the recorded response.

## Conclusions

The fecal microbiome of patients with SLE had lower bacterial richness and overall diversity compared to healthy controls, irrespective of medication use or disease severity. The characteristic microbial signature in female patients with SLE includes lower levels of *Agathobacter*,* Coprococcus*,* Ruminococcus*, and *Faecalibacterium*, alongside significantly higher levels of *Bacteroides* and *Escherichia-shigella.* Elevated *Prevotella* level in severe SLE cases suggests its potential as a severity biomarker.

## Electronic supplementary material

Below is the link to the electronic supplementary material.

**Additional File 1**: **Supplementary Table 1**. Systemic Lupus Erythematosus Disease Activity scoring system (SELENA-SLEDAI). **Supplementary Table 2**. Comprehensive data of patients with SLE. **Supplementary Table 3**. Number of Reads after quality and length filtering.

**Additional File 2**: **Fig. S1**. Rarefaction curves based on OTU count in healthy control and patients with systemic lupus erythematosus. **Fig. S2**. Gut microbial alpha diversity in patients with systemic lupus erythematosus with different disease severity and treatments. Alpha diversity estimated by different indices (Chao1, Observed OTUs, Shannon, and Simpson diversity indices) among (a) severe and mild/moderate systemic lupus erythematosus groups, (b) azathioprine treatment groups, (c) hydroxychloroquine treatment groups, (d) cyclophosphamide treatment groups, and (e) Prednisolone treatment groups stratified by dosage with low dose group receiving ≤ 10 mg/day and high dose group receiving ˃ 10 mg/day. Statistical analysis was performed using the Mann-Whitney test. *P* < 0.05 was significant. Error bars represent the standard deviation. **Fig. S3**. Comparative analysis of gut microbial taxa in patients with mild/moderate and severe systemic lupus erythematosus (SLE). Cladogram of Linear discriminant analysis effect size (LEfSe) of the microbiome of (a) patients with mild/moderate and severe SLE, (b) azathioprine-treated (AZA+) and untreated (AZA-) groups, (c) hydroxychloroquine-treated (HCQ+) and untreated (HCQ-) groups, and (d) cyclophosphamide-treated (CYC+) and untreated (CYC-) groups. Green and red circles are the significantly affected taxa. The diameter of each circle is proportional to the relative abundance of the taxon.

## Data Availability

The datasets generated and/or analyzed during the current study are available in the NCBI Sequence Read Archive (SRA) repository under Project Accession # PRJNA1038719 (SRA Experiments: SRR26819885 to SRR26819907), and the samples are assigned Serial Numbers: SAMN38198976 to SAMN38198985. https://www.ncbi.nlm.nih.gov/sra/?term=PRJNA1038719.

## Abbreviations

ACL: Anti-Cardiolipin
LPS: Lipopolysaccharide
ASV: Amplicon Sequence Variant
OTUs: Operational Taxonomic Units
AZA: Azathioprine
PCR: Polymerase Chain Reaction
BHIS: Brain Heart Infusion-Supplemented
PCoA: Principal Coordinate Analysis
CYC: Cyclophosphamide
PERMANOVA: Permutational Multivariate Analysis of Variance
DNA: Deoxyribonucleic Acid
PRED: prednisolone
ELISA: Enzyme-Linked Immunosorbent Assay
QIIME: Quantitative Insights Into Microbial Ecology
EDTA: Ethylenediaminetetraacetic Acid
rRNA: Ribosomal Ribonucleic Acid
HCQ: Hydroxychloroquine
SLE: Systemic Lupus Erythematosus
IL: Interleukin
SLEDAI: Systemic Lupus Erythematosus Disease Activity Index
IgG: Immunoglobulin G
Treg: Regulatory T Cells
IgM: Immunoglobulin M
UCG: Uncultured Genus Cluster
LEfSe: Linear Discriminant Analysis Effect Size
V3–V4: Variable Regions 3 to 4 of 16S rRNA gene
LBP: Lipopolysaccharide Binding Protein
