# Supplementary material for: Gut microbiota dysbiosis and associated immune response in systemic lupus erythematosus: impact of disease and treatment
Source: Gut Pathog. 2025 Feb 18;17:10. doi: 10.1186/s13099-025-00683-7 (PMC11834511; doi:10.1186/s13099-025-00683-7)
Supplement: Supplementary file 2 — Additional File 2: Fig. S1. Rarefaction curves based on OTU count in healthy control and patients with systemic lupus erythematosus. Fig. S2. Gut microbial alpha diversity in patients with systemic lupus erythematosus with different disease severity and treatments. Alpha diversity estimated by different indices (Chao1, Observed OTUs, Shannon, and Simpson diversity indices) among (a) severe and mild/moderate systemic lupus erythematosus groups, (b) azathioprine treatment groups, (c) hydroxychloroquine treatment groups, (d) cyclophosphamide treatment groups, and (e) Prednisolone treatment groups stratified by dosage with low dose group receiving ≤ 10 mg/day and high dose group receiving ˃ 10 mg/day. Statistical analysis was performed using the Mann-Whitney test. P < 0.05 was significant. Error bars represent the standard deviation. Fig. S3. Comparative analysis of gut microbial taxa in patients with mild/moderate and severe systemic lupus erythematosus (SLE). Cladogram of Linear discriminant analysis effect size (LEfSe) of the microbiome of (a) patients with mild/moderate and severe SLE, (b) azathioprine-treated (AZA+) and untreated (AZA-) groups, (c) hydroxychloroquine-treated (HCQ+) and untreated (HCQ-) groups, and (d) cyclophosphamide-treated (CYC+) and untreated (CYC-) groups. Green and red circles are the significantly affected taxa. The diameter of each circle is proportional to the relative abundance of the taxon. [file 13099_2025_683_MOESM2_ESM.docx]

***Gut Pathogens***

**Gut Microbiota Dysbiosis and associated Immune Response in Systemic Lupus Erythematosus: Impact of Disease and Treatment**

Aya Y. Ali^1^, Sara A. Zahran^1*^, Mervat Eissa^2^, Mona T. Kashef^3^, Amal E. Ali^1^

^1^ Microbiology & Immunology Department, Faculty of Pharmacy, Future University in Egypt, 12311, Cairo, Egypt.

^2^ Rheumatology & Rehabilitation Department, Faculty of Medicine, Cairo University, Cairo, Egypt.

^3^ Department of Microbiology and Immunology, Faculty of Pharmacy, Cairo University, Cairo, Egypt.

*** Corresponding author:**

Sara A. Zahran

Department of Microbiology& Immunology, Faculty of Pharmacy, Future University in Egypt, 12311, Cairo, Egypt

Email: [sara.zahran@fue.edu.eg](mailto:sara.zahran@fue.edu.eg)

**Supplementary Materials**


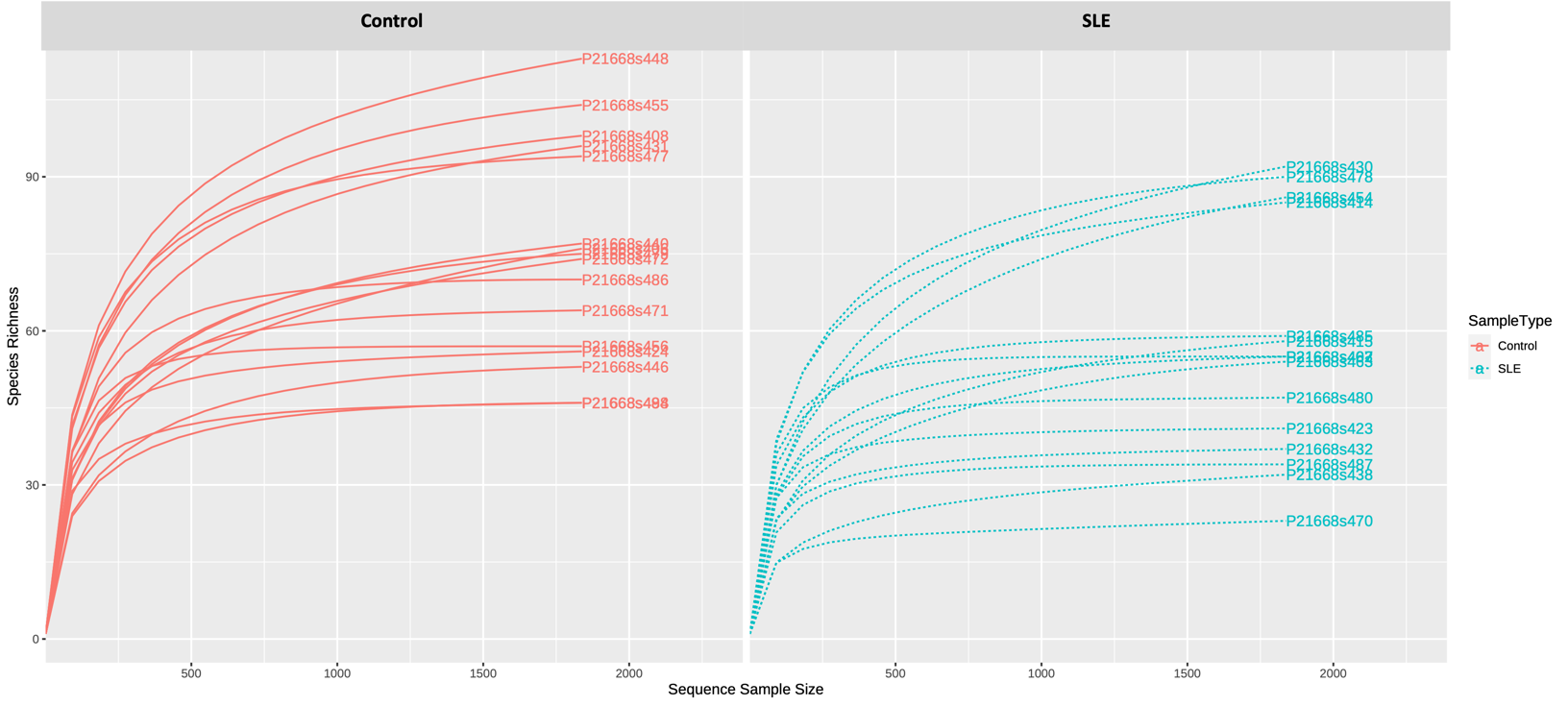


**Fig. S1** Rarefaction curves based on OTU count in healthy control and patients with systemic lupus erythematosus.


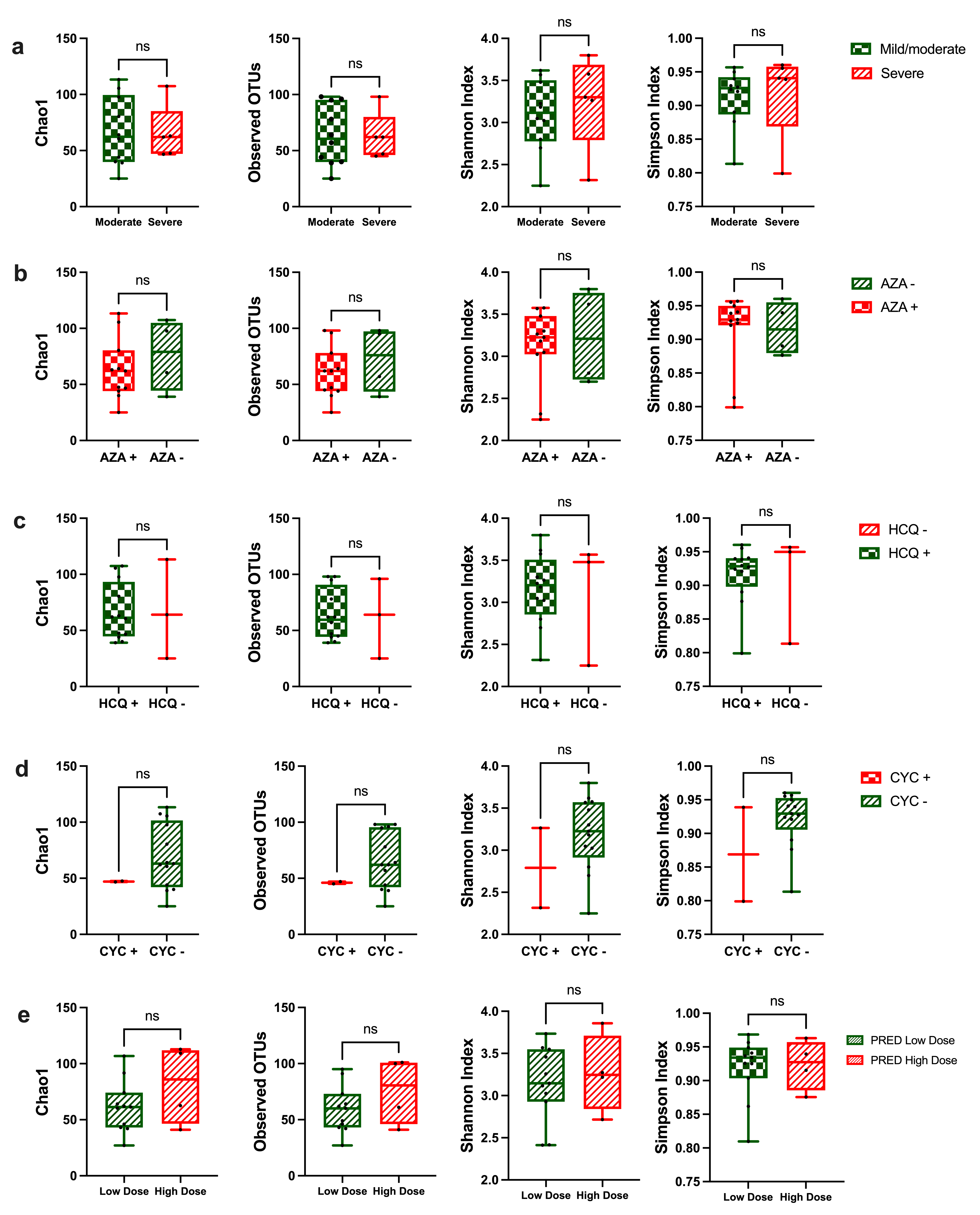


**Fig. S2 Gut microbial alpha diversity in patients with systemic lupus erythematosus with different disease severity and treatments**

Alpha diversity estimated by different indices (Chao1, Observed OTUs, Shannon, and Simpson diversity indices) among (a) severe and mild/moderate systemic lupus erythematosus groups, (b) azathioprine treatment groups, (c) hydroxychloroquine treatment groups, (d) cyclophosphamide treatment groups, and (e) Prednisolone treatment groups stratified by dosage with low dose group receiving ≤ 10 mg/day and high dose group receiving ˃ 10 mg/day. Statistical analysis was performed using the Mann-Whitney test. P < 0.05 was significant. Error bars represent the standard deviation.


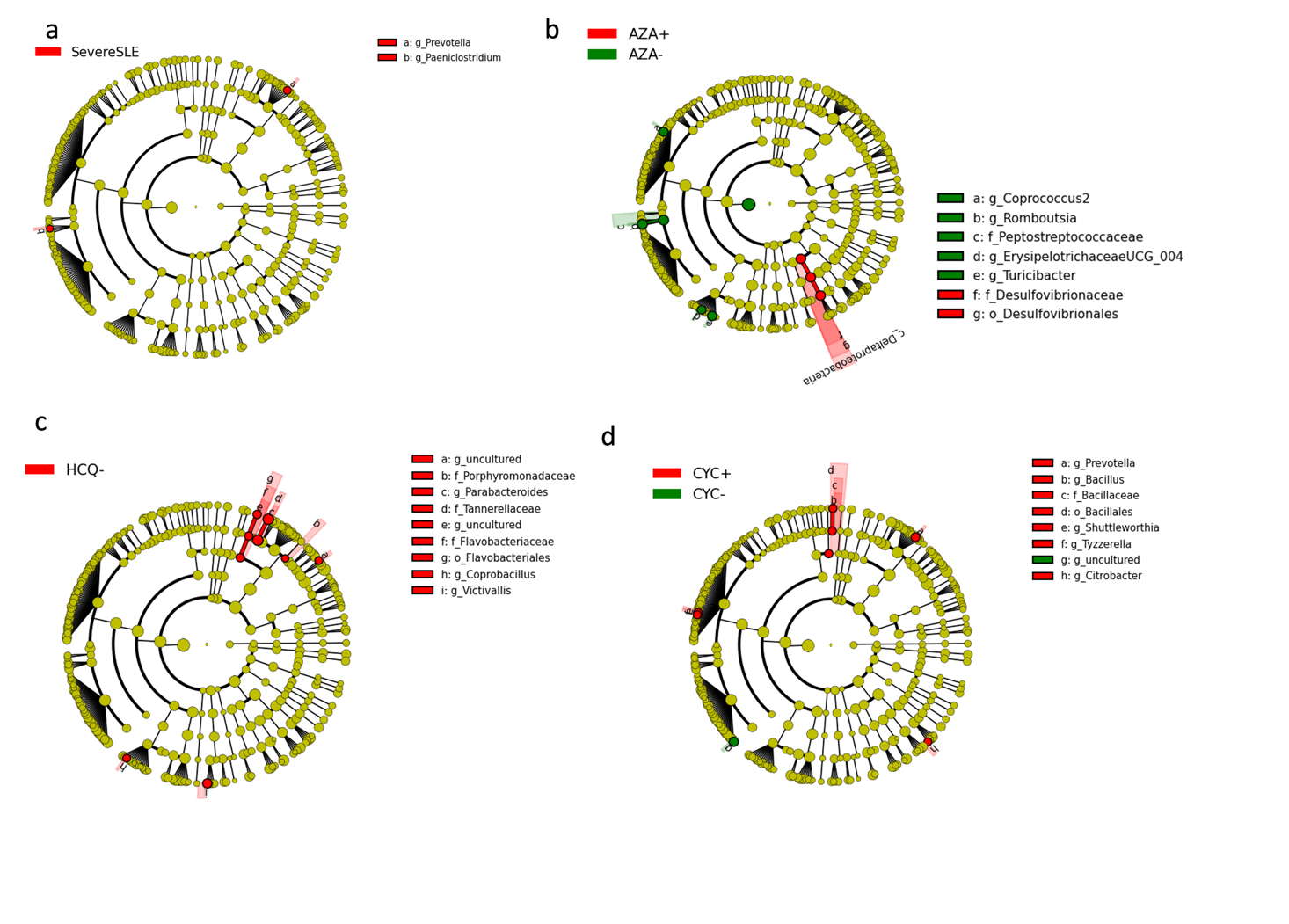


**Fig. S3 Comparative analysis of gut microbial taxa in patients with moderate and severe systemic lupus erythematosus (SLE)**

Cladogram of Linear discriminant analysis effect size (LEfSe) of the microbiome of **(a)** patients with moderate and severe SLE, **(b)** azathioprine-treated (AZA+) and untreated (AZA-) groups, **(c)** hydroxychloroquine-treated (HCQ+) and untreated (HCQ-) groups, and **(d)** cyclophosphamide-treated (CYC+) and untreated (CYC-) groups. Green and red circles are the significantly affected taxa. The diameter of each circle is proportional to the relative abundance of the taxon.
